# Formation of the oxidized flavor compounds at different heat treatment and changes in the oxidation stability of milk

**DOI:** 10.1002/fsn3.874

**Published:** 2018-11-13

**Authors:** Yan H. Li, Wei J. Wang, Fan Zhang, Zhi Peng Shao, Liang Guo

**Affiliations:** ^1^ College of Food Science and Biotechnology Engineering Zhejiang Gongshang University Hangzhou China; ^2^ Research & Development Institute Zhejiang Yi Ming Food Co. Ltd. Wenzhou China

**Keywords:** heat treatment, milk, oxidation stability, oxidized flavor compounds

## Abstract

The oxidized flavor could affect the sensory acceptability of consumers. In this study, the oxidized flavor compound (OFC) in different heated milk was analyzed using the solid phase microextraction–gas chromatography–mass spectrometry (SPME–GC–MS). The concentration of OFC increased with increasing the intensity of heat treatment. The concentrations of heptanal, nonanal, 2‐heptanone, and 2‐nonanone in the heated milk samples were in the range of 1.88–5.51, 1.03–3.26, 6.89–88.04, and 1.46–12.78 μg/kg, respectively. The correlation coefficients between the OFC and the heat intensity were above 0.86. It was found that the intensity of heat treatment (below 80°C for 10 min) could not cause the significant changes in the distribution of fat globules. The contents of partial proteins in milk fat globule membrane gradually destructed with increasing the intensity of heat treatment. The DPPH˙ scavenging activity reduced by 23.15%, and the peroxide value (POV) increased by 37.23% as raw milk was heated at 90°C for 20 min. Values of thiobarbituric acid reactive substances (TBARS) had a tendency to increase as the heated time was operated for 20 min (*p *<* *0.05). It indicated the heat treatment could change the oxidative environment of milk and influence the distribution of milk fat and the formation of oxidized flavor compounds.

## 1 INTRODUCTION

The flavor of milk depends on the aroma of the matrix. Fresh milk has a delicious flavor, which contains complex, diverse, and unstable ingredients (Bendall, 2001; Zhi, Zhao, & Shi, 2016). It is generally believed that the flavor of fresh milk is quite different due to different factors, such as cow itself, feed and feeding environment, lactation season, and so on. It had been reported that the flavor compounds in milk flavor involved free fatty acids, aldehydes, ketones, alkanes, esters, ethers, alcohols, lactones, sulfur compounds, terpenoids, and aromatic compounds (Nursten, 1997). These compounds had special flavor contribution in milk due to the different characteristics of diversity, instability, and thresholds.

Consumers’ acceptance or refusal of a certain food was mainly determined by its flavor (Nursten, 1997; Yeh, Schiano, Jo, Barbano, & Drake, 2017). The pleasant smell of consumers was aroma, and the odor which caused unpleasant feeling was off‐odor. The latter could be produced by absorbing the odor of feed, cattle barn, and the surrounding environment. The metabolism from bacterial contamination could make the milk produce the flavor, such as sour flavor, malt flavor or unclean taste. The chemical reactions resulted from the oxidation, light illumination, drugs, and spoilage also could contribute the different off‐odors in milk.

Heat treatment was an indispensable process in the production of dairy products. The heat intensity could be monitored by adjusting heating temperature or heating time. During the heating process, the flavor compounds and the flavor precursors in milk changed from different physicochemical reactions, which made the flavor compounds in the heated milk different from raw milk (Bendall, 2001; Contarini, Povolo, Leardi, & Piero, 1997). The main oxidized flavor precursor in milk was milk fat, and it was the key factor to decide the flavor of milk. Milk fat could be oxidized and decomposed during the process of milk heating, which resulted from the release of fatty acids and formed lots of flavor compounds from the main reactions of the automatic oxidation, dehydration, decarboxylation, reduction, and hydrolysis (Li & Wang, 2016; Li, Zhang, Wang, & Han, 2013; Nursten, 1997). Among the flavor compounds, the volatile aldehydes and ketones were the important components of the oxidized flavor in milk. In the previous studies, the aldehydes and the ketones were also evaluated as the main flavor compounds in the concentrated milk, distillate, and milk powder (Li & Wang, 2016; Li et al., 2013).

Compared with ultra‐high temperature (UHT) milk and milk powder, few studies focus on the flavor compounds in pasteurized milk. Contarini et al. (1997) pointed out that the concentrations of 2‐butanone, 2‐pentanone, toluene, hexanal, and 2‐heptanal in milk (heated at 78 °C for 15 s) were significantly different from the UHT milk (heated at 144 °C for 3.5 s) and bottled milk (heated at 120 °C for 30 min). Vazquez‐Landaverde, Velazquez, Torres, and Qian (2005) showed that the concentrations of 2‐hexanone, 2‐heptanone, 2‐nonanone, 2‐undecanone, 2‐methyl aldehyde, 3‐methyl butyral, heptanal, and decanal in UHT milk were higher than those in raw milk or in pasteurized milk. It also pointed out that levels of methyl ketones in UHT milk increased with the increase in fat content. Lloyd, Hess, and Drake (2009) reported that the concentrations of hexanal, 2‐heptanone, heptanal, octanal, nonanal, 3‐methyl aldehyde, and 2‐methyl aldehyde in milk powder increased significantly after storage of 12 months. The related research also pointed that the oxidized off‐odor occurred obviously as the concentration of hexanal was above 600 μg/kg or the concentration of nonanal was above 4 μg/kg in whole milk powder (Lloyd, Drake, & Gerard, 2009). Hall pointed that lipid oxidation influenced the flavor during storage of milk powder and straight‐chain aldehydes were the main flavor compounds, such as hexanal, heptanal, octanal, and nonanal (Hall & Andersson, 1985; Hall, Andersson, Lingnert, & Olofsson, 1985).

However, change in the oxidized flavor of heated milk was ignored although the flavor compounds would be quite different due to different heated intensity, which was essentially operated in the manufacture of low‐pasteurized milk, high‐pasteurized milk, or other dairy products. The natural fat globules in bovine milk are coated with a protective layer generally known as the milk fat globule membrane (MFGM). It markedly had the shielding effects on the milk lipid and could affect the lipid oxidation in milk (Mather, 2000). The study aimed at tracking the oxidized flavor compounds in different heated milk. It also explicated the destruction of milk fat as the main flavor precursor and the changes in the natural antioxidant capacity and oxidation susceptibility of different heated milk. It could provide the important theoretical and practical implications in the flavor quality of dairy products.

## 2 MATERIALS AND METHODS

### 2.1 Heating treatment

Raw milk (RM) obtained from a local dairy plant named XINGFU farm was added 0.02% (w/v) NaN_3_ to prevent bacterial growth. The milk sample was stored at 4–6°C. According to the previous methods (Li & Wang, 2016; Li et al., 2013), the heated milk (HM) was prepared with the heated intensity of 70, 80, and 90°C for 0.5, 2, 5, 10, 15, and 20 min respectively. The heated milk was cooled to ambient temperature (20 ± 1°C) in a stirred water bath at 0–4°C.

### 2.2 Milk component analysis

Milk fat was determined by the Rose–Gottlieb method and total protein by the Kjeldahl technique with a factor of 6.38 as described by the previous study (Guinee, Auty, & Fenelon, 2000). Total solids of RM were calculated according to the weight loss by drying the samples at 105 ± 1°C (Almeida, Tamime, & Oliveira, 2009).

### 2.3 Determination of volatiles

The volatiles in the headspace of the milk samples (raw milk, heated milk) were extracted and analyzed using SPME–GC–MS according to the previous studies (Li & Wang, 2016; Li et al., 2013). Heptanal, nonanal, 2‐heptanone, and 2‐nonanone, as selected oxidized volatiles, were identified by the NIST‐02L GC‐MS spectrum library and the retention time of their standard chemicals (Sigma, USA). The area of flavor compounds and internal standard could be provided by the GC‐MS. The concentrations of individual compounds were calculated using the area ratio of flavor compounds and internal standard. The odor activity value (OAV) of different volatiles was calculated by the ratio of the concentration and corresponding flavor threshold value. Only the volatile whose OAV was above 1.0 would be felt and contribute to the flavor (Qian & Reineccius, 2003).

### 2.4 Analysis of MFGM protein components

#### 2.4.1 Isolation and heating the MFGM material

Raw milk was heated at 70, 80, and 90°C for 2 min, respectively. After heating, the sample was cooled immediately to room temperature in the ice bath. Then milk sample was centrifuged at 15,000 *g* for 20 min at 20°C in a temperature‐controlled centrifuge (Sigma 3–30k, Germany), and the cream layer was removed and stored at 4°C. Then, the supernatant cream was washed with simulated milk ultrafiltrate (SMUF). The SUMF solution was prepared according to the literature (Lee & Sherbin, 2002; Le, Van Camp, Rombaut, van Leeckwyck, & Dewettinck, 2009). The above steps were operated for three times. The washed cream was resuspended in SMUF to yield a suspension with a fat content similar to that of the original milk.

#### 2.4.2 Determination of SDS‐PAGE electrophoresis

The individual proteins in the washed cream were determined by SDS‐PAGE with some modification of the reference (Ye, Singh, Oldfield, & Anema, 2004). The washed cream was dispersed (1:2 w/w) in 0.5 M Tris–HCl buffer, containing 10% glycerol, 2% (w/v) SDS, and 0.05% bromophenol blue. The gels were run in a Mini‐Protean Tetra Electrophoresis System (Bio‐Rad Laboratories, Hercules, CA, USA). The proteins of the MFGM were identified by comparing with the marker (Hercules, CA, USA).

### 2.5 Distribution of milk fat globules

Size and specific surface area (SSA) of fat globules in milk samples were determined using a particle size analyzer (ZetaPLAS, Brookhaven Instruments Corporation, USA) at 40°C water bath. The milk sample containing 2% (w/v) SDS and 50 mM EDTA was dispersed (Lee & Sherbin, 2002; Ye et al., 2004; Ye, Singh, Taylor, & Anema, 2002). The ratio of refractive index of fat globule was 1.456. Value of obscuration ranged from 8% to 10%. Running time was 60 s. Globule size was expressed as d_32_, and volume surface average diameter in μm. Average fat globule diameters were calculated in duplicate.

### 2.6 DPPH˙scavenging activity

The total antioxidative activity was determined by DPPH method (Li, Hosseinian, Tsopmo, Friel, & Beta, 2009; Smet et al., 2008) with some modification. 2,2‐Diphenyl‐1‐picrylhydrazyl (Sigma, USA) solution (200 μmol/L) was present in absolute methanol. Milk samples were mixed vigorously with DPPH solution (1:3, v/v) for 15 s. The mixture was centrifuged at 5,000 *g* for 10 min. The supernatant was held at 20°C for 20 min in a dark place.

### 2.7 Peroxide value

Peroxide value (POV) was used to analyze the oxidation of milk. POV analysis was based on the procedure of Smet et al. (2009).

### 2.8 Value of thiobarbituric acid reactive substances

Value of thiobarbituric acid reactive substances (TBARS) was determined based on the methods (Sun, 2013; Zhou, 2009).

### 2.9 Statistical analysis

Analyses were duplicated for the SPME–GC–MS expressed as mean, and triplicated expressed as mean ± *SD* for others. One‐way analysis of variance and univariate analysis of variance (ANOVA) for determining the effect of the processes were carried out using PASW Statistics 18.0 software (SPSS Inc, USA). Trend regression analysis was applied to analyze the parameters of oxidized volatiles by the intensity of heat treatment.

## 3 RESULTS AND DISCUSSION

### 3.1 Effect of heat treatment on the oxidized flavor of milk

#### 3.1.1 Detection of flavor compounds in milk

**Figure 1:**
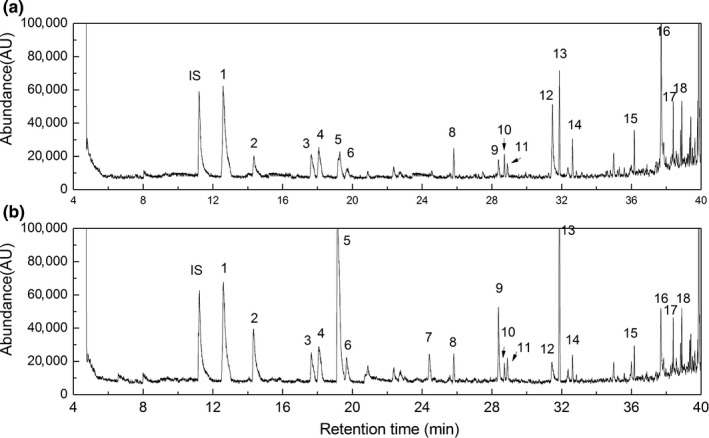
Total ion count chromatogram of volatiles derived from raw milk and heated milk. (a) Raw milk. (b) Heated milk (heated at 90°C for 15 min). Note: IS: internal standard; 1, toluene; 2, hexanal; 3, ethylbenzene; 4, p‐xylene; 5, 2‐heptanone; 6, heptanal; 7, decane; 8, limonene; 9, 2‐nonanone; 10, undecane; 11, nonanal; 12, octanoic acid; 13, 2‐methyl‐5‐[1‐methyl vinyl‐cyclohexanone]; 14, dodecane; 15, tridecane; 16, decanoic acid; 17, tetradecane; 18, ten hydrogen‐4,8,8‐three methyl‐9‐methylene‐1,4‐methoxyazulene

The average contents of the components in the milk samples were as follows: protein, 3.2 ± 0.1%; and fat, 3.2 ± 0.2%. Total solids of milk samples were 12.1 ± 0.3%. No difference was found in the compositions of raw milk and heated milk (*p *>* *0.05). Total ion count chromatograms of volatiles derived from raw milk and heated milk (heating at 90°C for 15 min) are shown in Figure 1. The flavor compounds in milk samples were quantified using the area ratio of volatiles and internal standard. Eighteen volatiles were identified from the samples, including three kinds of aldehydes, two kinds of ketones, two kinds of acids, five kinds of hydrocarbons, three kinds of benzenes, and three kinds of other compounds.

Compared with the previous studies, Hettinga, van Valenberg, and van Hooijdonk (2008) identified seven compounds in raw milk using SPME–GC–MS (acetone, dimethyl sulfide, carbon disulfide, 2‐butanone, chloroform, pentanal, and hexanal), while six compounds (acetone, dimethyl sulfide, 2‐butanone, chloroform, pentanal, and hexanal) were identified using purge and trap–gas chromatography–mass spectrometry (PT–GC–MS). Hougaard, Vestergaard, Varming, Bredie, and Richard (2011) found there were 12 kinds of flavor compounds using PT–GC–MS, while Contarini and Povolo (2002) found nine kinds of flavor compounds using SPME–GC–MS in pasteurized milk. The types of flavor compounds found in this study differed from those with the previous reports, which probably reasoned from the factors of cow's lactation, environment, processing operation, and the identified methods. Compared with the raw milk, the concentrations of ketones and aldehydes in the heated milk increased significantly (*p *<* *0.05). Volatiles of heptanal, nonanal, 2‐heptanone, and 2‐nonanone were analyzed as the main oxidized flavor compounds in this study.

#### 3.1.2 Changes in oxidized flavor compounds

**Figure 2:**
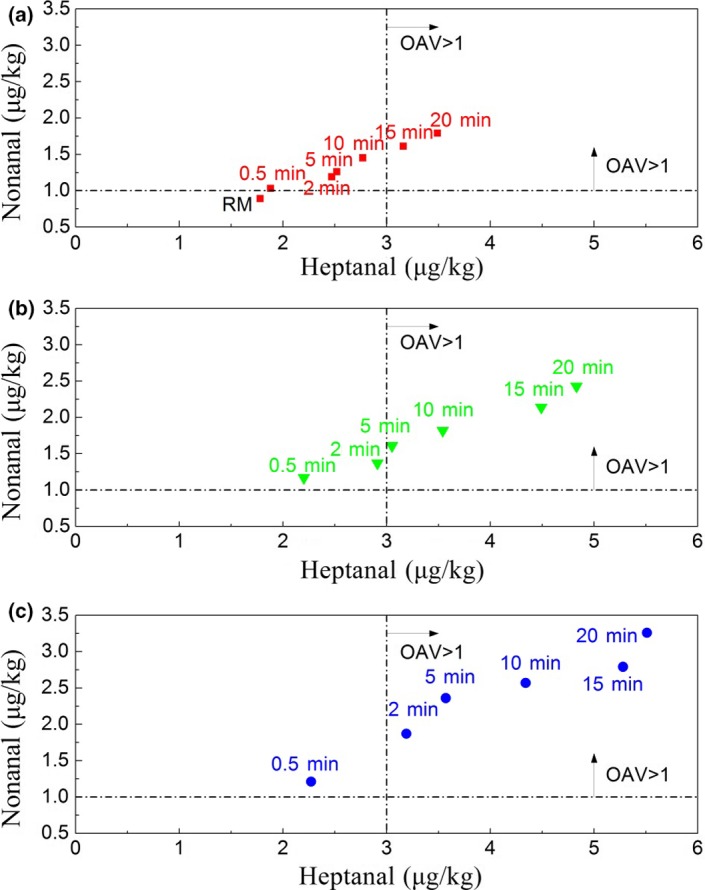
Levels of heptanal and nonanal in different heated milk. RM: raw milk; OAV: odor activity value. (a) Heated milk group at 70°C. (b) Heated milk group at 80°C. (c) Heated milk group at 90°C

Levels of heptanal and nonanal in raw milk and heated milk are shown in Figure 2, which increased with increasing the heating intensity. Concentration of heptanal in the heated milk at 90°C for 5 min was 3.57 μg/kg and that was different in the heated milk at 70°C for 5 min (2.52 μg/kg) (*p *<* *0.05). When the heating intensity was operated at 90°C for 20 min, the concentration of heptanal was 5.51 μg/kg. The concentration of nonanal in the milk samples ranged from 1.03 to 3.26 μg/kg. Heptanal and nonanal in raw milk or heated milk could be felt according to the corresponding thresholds in milk (Vazquez‐Landaverde, Torres, & Qian, 2006). That indicated heptanal and nonanal in the heated milk showed flavor contribution significantly and endowed milk samples with fatty flavor and fatty smell, respectively.

**Figure 3:**
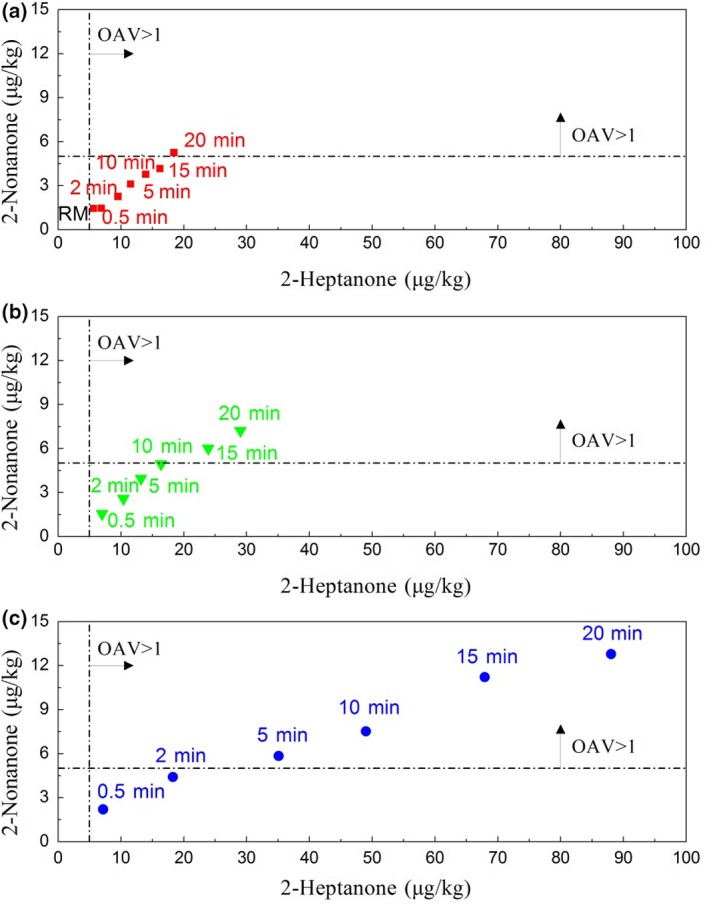
Levels of 2‐heptanone and 2‐nonanone in different heated milk. RM: raw milk; OAV: odor activity value. (a) Heated milk group at 70°C. (b) Heated milk group at 80°C. (c) Heated milk group at 90°C

Figure 3 shows levels of 2‐heptanone and 2‐nonanone in different heating milk. Concentration of 2‐heptanone increased with increasing the heat intensity obviously, which ranged from 6.89 to 88.04 μg/kg in the milk samples, respectively, in the 90°C heating group. Concentration of 2‐nonanone ranged from 1.43 to 12.78 μg/kg among the heated milk. It was significantly different for the 90°C heated groups and the 70°C heated groups (*p *<* *0.05). The OAV of 2‐heptanone was 1.13 in raw milk according to the threshold in milk (Vazquez‐Landaverde et al., 2006). Fresh cow milk has a distinctive, and yet subtle, delicate flavor (Bendall, 2001). It indicated that 2‐heptanone had important flavor contribution as the basic flavor component of milk sample. However, 2‐nonanone had no flavor contribution because its OAV was less than 0.5 in raw milk (the threshold was 5 μg/kg in milk) (Vazquez‐Landaverde et al., 2006). The result of 2‐nonanone in raw milk was consistent with the previous studies (Hougaard et al., 2011).

The flavor contribution of the methyl ketones improved with increasing the heat intensity obviously. OAV of 2‐heptanone was 17.61 as the heating intensity reached to 90°C for 20 min. OAV of 2‐nonanone was above 1.0 as heating intensity reached to 70°C for 20 min, 80°C for 15 min, or 90°C for 5 min. Those endowed the heated milk with soap‐oxidized flavor and grassy‐oxidized flavor, respectively.

#### 3.1.3 Dependency relationship of OFC and the heating intensity

The dependency relationship between the concentration of OFC and the regression function of heating temperature and time is shown in Table 1. A single parameter of oxidized flavor compound (*z*, including heptanal, nonanal, 2‐heptanone, and 2‐nonanone) was used as the dependent variable. Heating temperature (×°C) and heating time (*y*, min) and their quadratic term respectively were used as independent variables to get the dependency relationship. The results showed that two quadratic polynomial could increase the determination coefficient *R* ^2^. The stepwise regression method was used to determine the model, and F value was less than 0.05 as the standard. The coefficient of determination was 0.868–0.936, which indicated that the prediction could explain the relationship between heating intensity and the corresponding concentration of OFC in the heated milk. The increase of methyl ketone was related to the β‐oxidation of β‐hydroxyl fatty acids and to the decarboxylation of β‐keto acids in the saturated fatty acids. The increase of aldehydes was related to the oxidation of unsaturated fatty acids (Nursten, 1997).

**Table 1:** Trend regression analysis on the oxidized volatiles by the intensity of heat treatment^a^

Independent variables	Prediction variable model^b^	Coefficient ahead of term	Coefficient of determination (*R* ^2^)
Heptanal	Constant	0.205	0.884
	*x*	0.027	
	*y*	−0.232	
	*xy*	4.38 × 10^−3^	
Nonanal	Constant	0.238	0.868
	*y*	−0.140	
	*x* ^*2*^	1.62 × 10^−4^	
	*xy*	2.52 × 10^−3^	
2‐Heptanone	Constant	8.304	0.869
	*y*	−12.212	
	*xy*	0.176	
2‐Nonanone	Constant	0.111	0.928
	*y*	−1.045	
	*x* ^*2*^	3.24 × 10^−4^	
	*xy*	0.017	

a Stepwise regression.

b *p *<* *0.001.

### 3.2 Effect of heat treatment on the precursor of oxidized flavor

#### 3.2.1 Changes in the MFGM proteins

**Figure 4:**
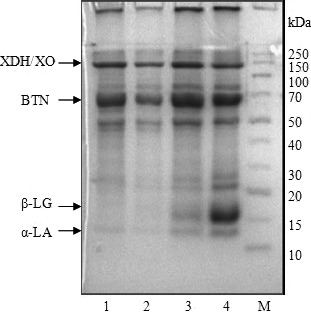
SDS‐PAGE patterns of the MFGM proteins for raw milk and heated milk. Note: M: marker; lanes 1–4 represent the MFGM protein samples of raw milk and heated milk samples prepared with the heat treatments at 70, 80, and 90°C for 2 min, respectively

Figure 4 shows the SDS‐PAGE result of MFGM proteins isolated from raw milk and the heated milk. The MFGM protein bands from the gel electrophoresis were Mucin1 (MUC1, 172.8–257.2 kD), xanthine dehydrogenase/oxidase (XDH/XO, 137.9–143.0 kD), periodic acid–Schiff IV (PAS IV, 75.8–78.6 kD, Butyrophilin (BTN, 63.8–66.3 kD), periodic acid–Schiff 6/7 (PAS6/7, 45.0–47.6 kD), and some unknown proteins (17.8–30.4 kD) (Lopez, 2011; Mather, 2000). It showed that the composition of MFGM protein changed gradually with the increase in heating intensity. The bands of β‐lactoglobulin (β‐LG) and α‐lactalbumin (α‐LA) appeared from the isolate of MFGM proteins, and the protein bands were the deepest at 90°C. That means the MFGM proteins could react with β‐LG and α‐LA as raw milk was heated above 80°C. The reaction probably resulted from the intermolecular and intramolecular thiol reactions among XDH/XO, BTN, β‐LG, α‐LA in the milk system (Ye et al., 2002, 2004). On the other hand, the polypeptides existed in MFGM protein contained a large number of cysteine residues, which might also participate in the reactions with whey protein as milk was heated (Kim & Jimenez‐Flores, 1995). That could decrease the shielding effects of MFGM protein and cause the accumulation of fat particles.

#### 3.2.2 Sizes distribution of milk fat globules

**Figure 5:**
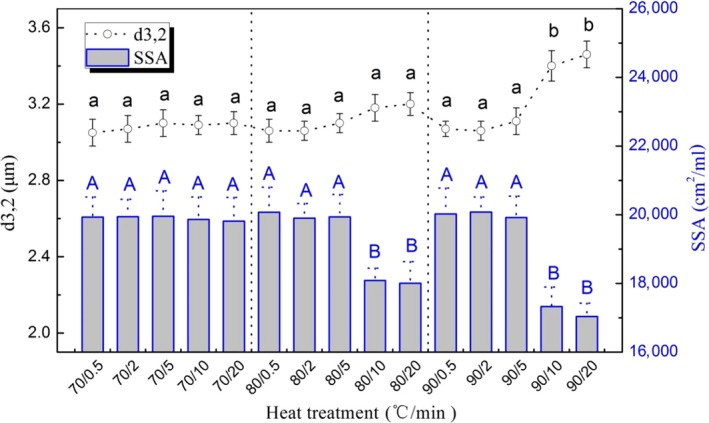
Difference in diameter (*d*_3,2_) and specific surface area (SSA) of fat globules among heated milk. Note: Bars with different letters were significantly different from each other (*p *<* *0.05)

**Figure 6:**
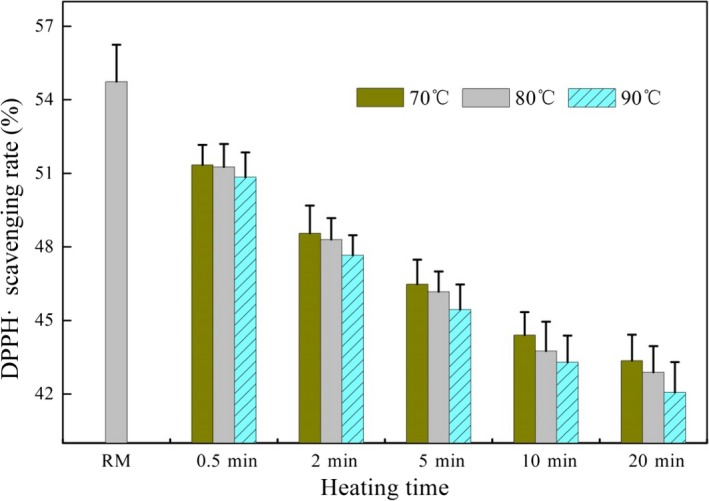
DPPH˙ scavenging activity of different heated milk

The diameter of fat globules in raw milk ranged from 1.15 to 7.42 μm along with a slightly normal distribution (data was not shown). Values of skewness and kurtosis were 0.3991 and −0.3971, respectively. Compared with the previous studies, it was indicated that the diameter of milk fat globule ranged from 0.1 to 10 μm and from 1.0 to 10 μm, respectively (Le et al., 2009; Lee & Sherbin, 2002). In this study, the diameter (*d*_3,2_) of the milk fat globules in different heated milk is shown in Figure 5. It was not significantly different among the group of raw milk (*d*_3,2_, 3.04 μm) and the group of heated milk except the samples heated at 90°C for 10 min and for 20 min (*p *>* *0.05). The specific surface area of milk fat globules among different milk samples is also shown in Figure 6. The SSA was from 19,815 to 19,959 cm^2^/ml after heating at 70°C for 0.5–20 min (*p *>* *0.05). After heating at the relative high heating intensity, such as heating at 80°C or 90°C for 10 min or 20 min respectively, the SSA of milk fat globules in the heated milk decreased significantly (*p *<* *0.05).

Raikos, Kapolos, Farmakis, Koliadima, and Karaiskakis (2009) found that the average size of milk fat globule was 0.861 μm as the whole milk was homogenized at 50°C and the size was up to 0.901 μm after heating at 125°C (*p *<* *0.05). It was contradicted by the previous result that heat treatment could not change the size distribution of milk fat (van Boekel & Folkerts, 1991). In this study, the diameter (*d*_3,2_) of the milk fat globules increased significantly as the heating intensity raised to 90°C for 10 min (*p *<* *0.05). The analysis of size distribution as affected by different heating treatments is shown in Table 2. That also indicated the low intensity of heating treatment (heating temperature was below 70°C or heating time was below 5 min) could not change the diameter (*d*_3,2_) and SSA of milk fat globules in milk (*p *>* *0.05). As the raw milk was heated above 80°C, the heat treatment influenced the distribution of diameter (*d*_3,2_) and SSA significantly and the *p* values were 0.046 and 0.002, respectively. On the other hand, it was effective as heating time prolonged above 10 min (*p *<* *0.003).

**Table 2:** Results of sizes distribution as affected by different heating treatments

Heat treatment	Effectiveness/P	
	*d*_3,2_	SSA
Heat temperature (°C)		
70	0.879	0.998
80	0.046	0.002
90	0.000	0.000
Heat time (min)		
0.5	0.962	0.967
2	0.982	0.881
5	0.981	0.997
10	0.003	0.003
20	0.001	0.003

### 3.3 Effect of heat treatment on milk oxidation stability

#### 3.3.1 Determination of the DPPH˙ scavenging activity in milk

Figure 6 shows the DPPH˙ scavenging activity of raw milk and the heated milk. The DPPH˙ scavenging activity of raw milk was 54.74%, which reasoned from the antioxidants, such as fat‐soluble vitamin A and vitamin E, transition metal ions, and water‐soluble vitamin C and vitamin B (Soberon, Liu, & Cherney, 2012). The value of DPPH˙ scavenging activity in raw milk was close to the previous study, which showed that the ability of raw milk to remove DPPH was 53% (Liu, Chen, & Lin, 2005). Compared with the raw milk, DPPH scavenging activity in the heated milk had the trend to decrease. Heating temperature had little effect on the DPPH˙ scavenging activity, and the difference was not significant in the temperature groups (*p *>* *0.05). It decreased continuously with the prolongation of heating time. As the raw milk was heated at 70, 80, and 90°C for 0.5 s, the DPPH˙scavenging activity decreased 6.23%–7.12%. It decreased 20.79%–23.15% after the raw milk was heated at 70, 80, and 90°C for 20 min. The loss of total antioxidant capacity of milk mainly resulted in the decrease of heat sensitive antioxidant. It could be concluded that reducing heating intensity during the process of heat treatment, especially shortening heating time, could reduce the decrease of DPPH˙ scavenging activity in milk system to control the oxidative environment and reduce the formation of oxidized flavor compounds.

#### 3.3.2 Peroxide value and TBARS in the heated milk

**Figure 7:**
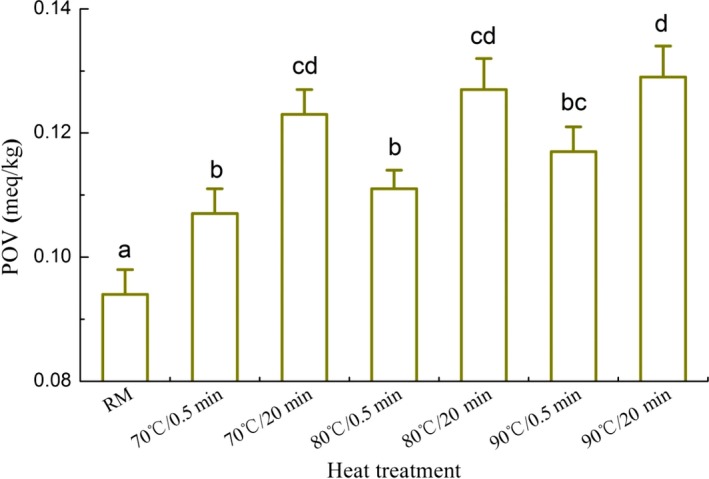
Peroxide value (POV) of different heated milk. Note: Bars with different letters were significantly different from each other (*p *<* *0.05)

**Figure 8:**
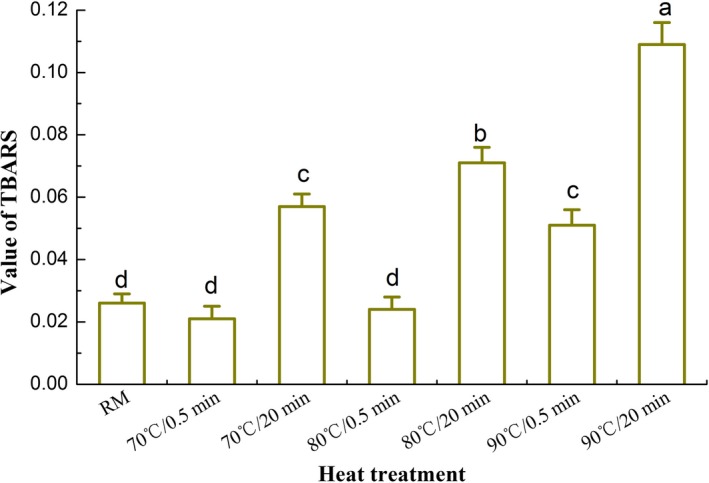
Value of thiobarbituric acid reactive substances (TBARS) of different heated milk. Note: Bars with different letters were significantly different from each other (*p *<* *0.05)

Levels of POV in different heated milk are shown in Figure 7. POV of raw milk was only 0.094 meq/kg. As it was heated at 70, 80, and 90°C for 0.5–20 min, values of POV increased significantly (*p *<* *0.05) and ranged from 0.107 to 0.129 meq/kg, which could indicate the oxidation of milk fat. During the course of milk heating, the formation of OFC was probably related to the oxidation of milk fat. It suggested the heat treatment could increase POV and the intermediate product of lipid oxidation could produce the final oxidized flavor compounds. Levels of TBARS in different heated milk are shown in Figure 8. Although values of TBARS in heated milk were very low, that had a tendency to increase as the heated time was operated for 20 min (*p *<* *0.05). In theory, the reactions among the MFGM proteins could decrease the shielding effects and cause the accumulation of milk fat particles in high heated milk. The sizes of the milk fat globules increased and the SSA of fat globules decreased consequently. As a result, the free fat is formed in milk and the values of POV and TBARS increased following the formation of oxidized flavor. These result indicated the distribution of milk fat during the course of heating could influence the formation of oxidation flavor.

## 4 CONCLUSIONS

Levels of typical oxidized flavor compounds in the heated milk increased with increasing the heating intensity. The determination coefficient between volatiles of heptanal, nonanal, 2‐heptanone, and 2‐nonanone with the heating intensity ranged from 0.868 to 0.936 at the given heating conditions. MFGM proteins extracted from the heated milk had reacted with whey proteins as heating temperature was above 80°C. Heat treatment also could increase the diameter and decrease the SSA of milk fat globules. That suggested heat treatment could decrease the shielding effects of MFGM protein and cause the accumulation of fat particles. Compared to raw milk, the DPPH˙ scavenging activity decreased and values of POV increased as prolonging heating time. It indicated heat treatment could influence the distribution of milk fat and increased the formation of the oxidized flavor compounds.

## ETHICAL STATEMENT

There is not any human or animal testing involved in this study.
